# Management of Complex Pelvic Ring and Acetabular Fracture Associated With Open Tibia Fracture in Severe Polytrauma Patient: A Case Report

**DOI:** 10.7759/cureus.21356

**Published:** 2022-01-18

**Authors:** Povilas Masionis, Tomas Vileikis, Petryla Giedrius, Igoris Šatkauskas, Valentinas Uvarovas, Giedrius Kvederas

**Affiliations:** 1 Clinic of Rheumatology, Orthopedic Traumatology and Reconstructive Surgery, Faculty of Medicine, Vilnius Republican University Hospital, Vilnius, LTU

**Keywords:** crush syndrome, open fracture, polytrauma patient, acetabular fractures, pelvic ring injury

## Abstract

With the increase of high energy injuries, acetabular and pelvic ring fractures tend to be a more common part of polytrauma patients. Despite growing incidence, management of these injuries remains one of the most difficult challenges in orthopedic surgery. As these patients are usually multiply injured, it is not only life-threatening trauma in acute settings but also crippling in long time.

We present a case of a 40-year-old male who suffered from a dreadful traffic accident following the fractures of pelvic ring and both acetabulum, open fracture of tibia, urinary bladder rupture, and crush syndrome. We discuss the factors which dictated the timing of definitive management of pelvic and associated injuries and compare it with this study. Furthermore, we present patients' final outcomes and management of long-term complications.

## Introduction

Acetabular and pelvic ring fractures remain major challenges in orthopedic surgery. Because of the curve of learning, only large reference centers are capable of managing these fractures [1]. With the increase of high energy injuries, acetabular and pelvic ring fractures tend to be a common part of polytrauma patients. Up to 61.7% of all patients with pelvic ring injuries are multiply injured and 12.2% of such patients have a concomitant soft tissue injury [2]. The mortality rate of pelvic ring fracture tends to be from 7% for an isolated injury to 31.1% when it is associated with severe damage to soft tissues [2,3]. The mortality rate also increases to 13%, when the fracture of pelvic ring is associated with acetabular fracture [4]. Therefore, choosing the right and timely handling with appropriate resuscitation, especially for polytrauma patients, is a heavyweight decision. Despite the success in acute period, it is even more difficult task to predict the function of salvaged limb and hip joint in the future.

Herein, we report a 40-year-old male who suffered from a dreadful motor vehicle accident following the fractures of pelvic ring and both acetabulum, open fracture of left tibia, urinary bladder rupture, and crush syndrome. We discuss challenges and factors which impacted timing of definitive management of acetabular fracture and soft-tissue defect in acute phase and final outcome with the need of total hip arthroplasty (THA). We also reviewed the pertinent literature about the prognostic factors for hip joint function following the fixation of acetabular fracture, the role of systemic inflammatory response syndrome in timing, and Vacuum-assisted closure device (VAC system {San Antonio, TX: Kinetic Concepts, Inc.}) in management of soft tissue defects [1-4].

## Case presentation

**Figure 1:**
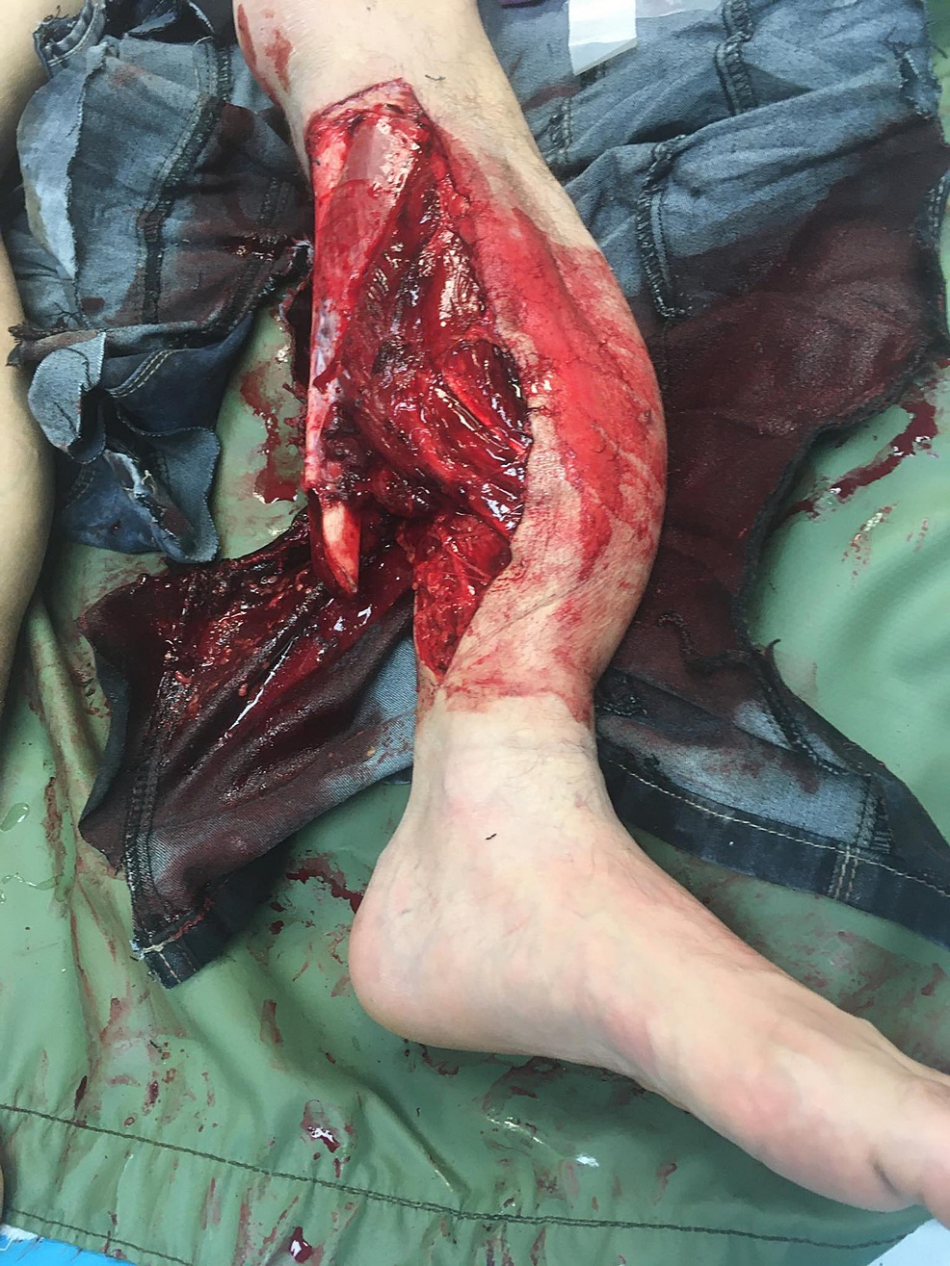
Open injury of the diaphysis of left shin bones (type 3B according to the Gustilo-Anderson classification).

A 40-year-old male pedestrian was presented to the emergency department with polytrauma after being hit by a vehicle. After the examination of the patient according to the local polytrauma protocol, full-body computed tomography angiography (CTA) was performed. The overall examination revealed an open injury of the diaphysis of left tibia (type 3B as per the Gustilo-Anderson classification [5]) (Figure 1), left side pelvic ring fracture (type B1.1 as per the Orthopaedic Trauma Association {OTA} classification [6]), transverse undisplaced fracture of the right acetabulum, left acetabular transverse + posterior wall fracture (as per the Judet and Letournel classification) with posterior dislocation of the hip, crush syndrome, and urinary bladder rupture.

**Figure 2:**
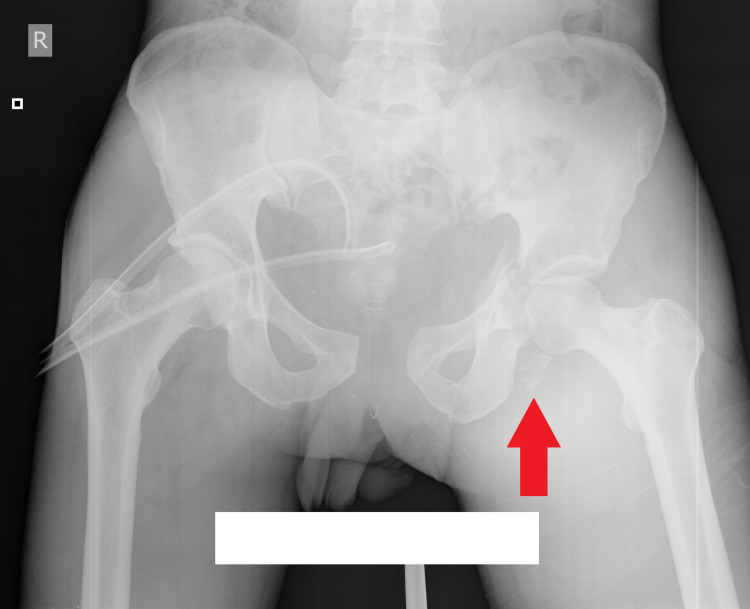
X-ray after closed reduction of left hip joint. The arrow shows markedly displaced posterior wall of acetabulum. Left side pelvic ring fracture (type B1.1 according to the Orthopaedic Trauma Association {OTA} classification), transverse undisplaced fracture of the right acetabulum, left acetabular transverse + posterior wall (according to Judet and Letournel classification) are present.

Despite that CTA had not shown active internal bleeding or brain injury, the patient was hemodynamically unstable and unconscious from the start of the admission. Immediately, the patient underwent open repair of bladder rupture, debridement of left thigh and external fixation by linear fixator, and closed reduction of left hip (Figure 2).

**Figure 3:**
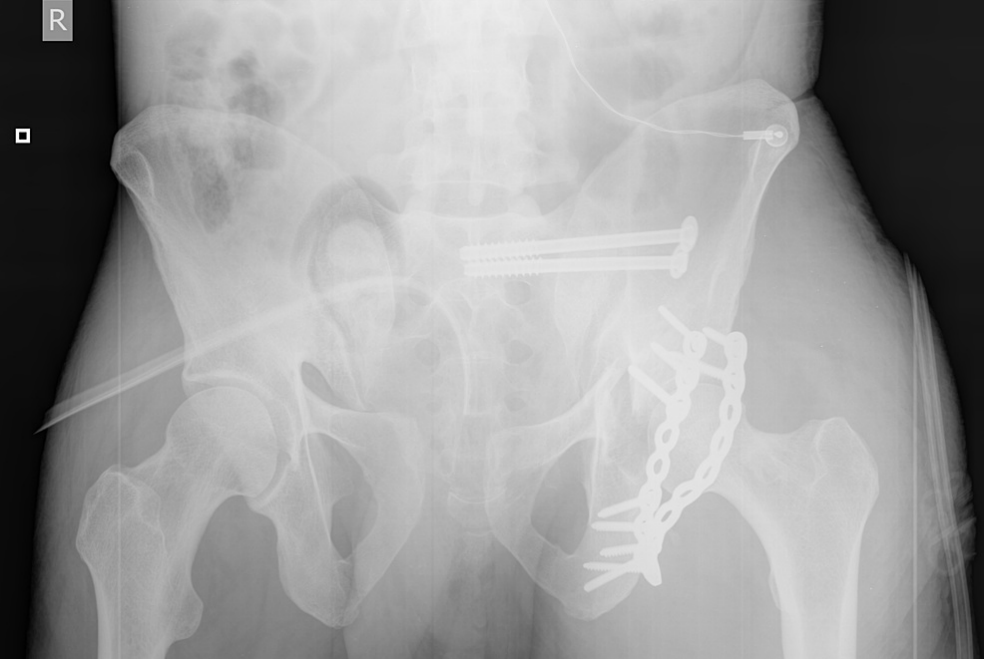
ORIF of the posterior column of the left acetabulum and CRIF fixation of left side sacroiliac joint. ORIF: open reduction and internal fixation; CRIF: closed reduction and internal fixation

Skeletal traction was applied to left hip joint because it was highly unstable. From the third day, patient required hemodialysis because of kidney failure and the palsy of tibial and peroneal nerves were present. Furthermore, elevated inflammatory markers and fever up to 39°C were present, which was an expression of systemic inflammatory response syndrome (SIRS) caused by trauma. Until the resolution of the syndrome, definitive management of pelvic fracture and soft tissue defect was postponed; the vacuum-assisted closure (VAC system) and repeated debridement of the wound on third, fifth, ninth, and 13th day were carried out. On 13th day after the accident, open reduction and internal fixation (ORIF) of the posterior column of the left acetabulum (through the Kocher-Langenbeck approach) and closed reduction and internal fixation (CRIF) of left side sacroiliac joint were performed (Figure 3).

**Figure 4:**
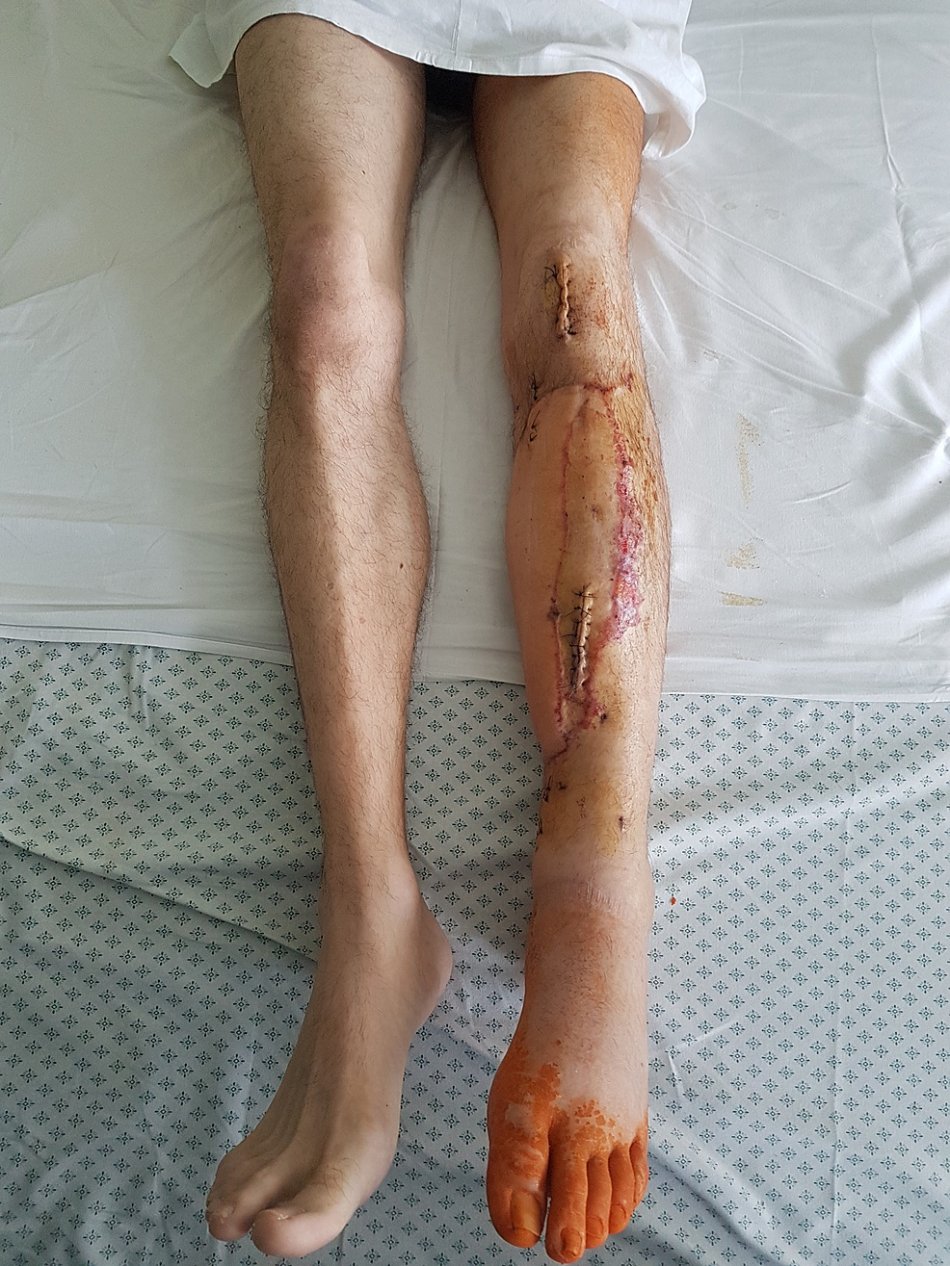
Healed soft tissues of left tibia after a latissimus dorsi pedicle flap reconstruction.

Syndesmosis and anterior column were not fixed because of the previous bladder injury. We did not use external fixator for the anterior pelvic ring, because sacroiliac joint fixation provides sufficient stability in type B1 fractures. Excessive soft tissue damage was found: complete rupture of gluteus maximus muscle belly and gluteal fascia, ruptures of external rotators of hip, and necrosis of abductor minimus muscle. Furthermore, fragment of the posterior wall of acetabulum was found dislocated distal to calcar of femur (Figure 2). The following day, extubation of the patient was followed by delirium, which took five days to resolve. On 20th day after the accident, reconstruction of soft tissues of tibia using a latissimus dorsi pedicle flap was performed (Figure 4).

**Figure 5:**
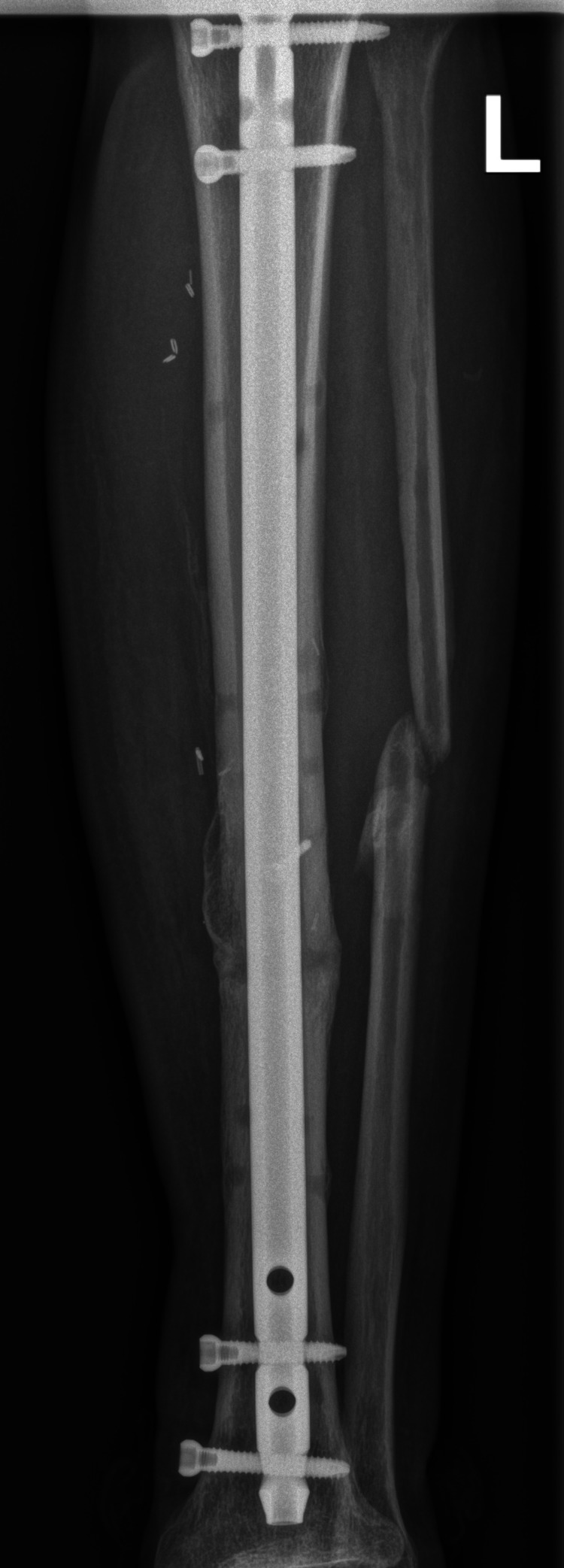
Complete union of left tibia after intramedullary nailing with iliac crest bone graft.

In the following few weeks, linear external fixator was exchanged to Ilizarov fixator. At four months, there were no signs of tibia healing and the external fixator was exchanged to intramedullary locking nail with bone grafting from iliac crest. At seven months, the patient regained the ability to walk without any assistance and there was full healing of left tibia (Figure 5). But 13 months after the accident, he presented to the clinic with a severe left hip pain - six points on visual analog scale, pain in left inguinal area during physical activity, an inability to step on his left leg, and a feeling of its shortening.

**Figure 6:**
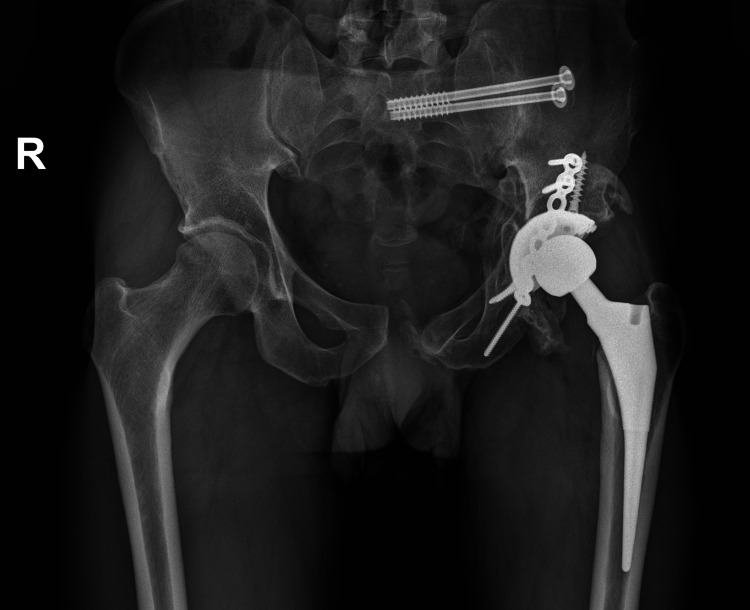
Uncemented THA of the left hip joint. THA: total hip arthroplasty

After a CT assessment, we diagnosed avascular necrosis of the femoral head and posterior wall of acetabulum (Video 1), therefore an uncemented THA through posterolateral approach was performed, using Taperloc stem and Continuum acetabular system (Warsaw, Indiana: Zimmer Biomet) (Figure 6).

**Video 1:** CT scan shows avascular necrosis of left femoral head and posterior wall of acetabulum.

Further postoperative period was uneventful. At 18 months after the initial trauma, the patient was able to walk short distances without any aid, for longer distances he used single left crutch (Video 2). The function of tibial and peroneal nerve improved, although paresis of the left foot remained. The patient's hip disability and osteoarthritis scores at the final follow-up were pain 75, other symptoms 50, function in daily activity 84, function in sport and recreation 83, and hip-related quality of life 50.

**Video 2:** Patient is able to walk short distances without any aid 18 months after the accident.

## Discussion

Complex pelvic fractures are one of the most deadly and devastating injuries which are usually not isolated [7]. Physiological status of the patient is the main indicator for definitive management of pelvic fractures, although it is a great challenge to time management of associated injuries. It is well known that early closure of Gustilo-Anderson type 3 fractures is associated with lower rates of infection and non-union. Some authors advocate early closure with local or pedicle flap within 24 hours, but it is generally accepted that closure is preferable within 72 hours and should not exceed five to seven days [8]. Our case is a perfect example of a patient who is unfit for early complex reconstructive surgery because of a general condition and a highly unstable hip joint. In this case, VAC therapy serves as early temporary closure of the wound. Recently, negative wound pressure therapy suffers substantial criticism in randomized controlled trials (RCTs) as being expensive and of low availability as compared to conventional wound dressing in open fractures treatment [9]. Because of this reason, we could not recommend it for use on a daily basis. Although in our case, cultures were taken at the time of every debridement and all of them were negative, in our opinion, VAC therapy is valuable option in preventing wound colonization and lowering the risk of surgical site infection when definitive treatment of one segment takes part before the soft-tissue defect is closed. Furthermore, it is not necessary to have an expensive VAC system as it is possible to build one from common hospital materials [10].

Another hazard encountered on the first few days after the injury that delayed the definitive management of pelvic fracture was the development of SIRS. According to the literature, in multiple trauma patients, SIRS usually begins on the first day and settles down after four days. Moreover, roughly 10 days after polytrauma develops, the compensatory anti-inflammatory response syndrome (CARS) reduces the immune defenses of the patient against the surgery [11]. Therefore, ideally, the patient should undergo surgery either on the first day or approximately between the fourth and 10th days after the trauma. The decision should be made taking into account the systemic homeostasis stability of the patient. Stable patients with a temperature of above 34°C, the absence of coagulopathy (fibrinogenemia above 1 g/L), and an absence of acidosis (pH above 7.2) may benefit from early definitive management immediately after presentation to the emergency department [12].

The present case demonstrates posttraumatic avascular necrosis of femoral head and posterior wall of acetabulum, which developed one year after ORIF and required THA. In the literature, the causes of avascular necrosis of acetabulum after the fracture of posterior column are well documented, the main causes for it being high-velocity trauma leading to excessive injury of soft tissues, fracture fixation with wide approach and excessive soft tissue stripping, marked fracture displacement or comminution, and delayed time between the surgical procedure and the time of injury [13]. All of them appeared in the present case and avascular necrosis was well expected, but we would not recommend early THA instead of ORIF in young patients because of the following reasons: (1) we should always opt for the salvage of the joint in young person and (2) it is better to wait until pelvic discontinuity is healed and use primary THA implants instead of revision systems. Although primary THA after acetabular fracture is the treatment of choice in older patients [14].

## Conclusions

Complex pelvic fractures in multiply injured patients are one of the deadliest in acute settings and one of the most crippling injuries in long term. The right and timely handling with appropriate resuscitation is the key in the management of these injuries. The definitive management of pelvic fracture is mainly dictated by the patient's physiological status and selected cases might benefit from immediate internal fixation, although, if postponed, it should take place not earlier than four days after the trauma. Furthermore, left open fracture is not a contraindication for internal fixation of pelvic fracture as VAC systems could be used as a temporary closure measure. Avascular necrosis of femoral head after acetabular fracture is a common sequela and joint salvage in a young person is mandatory, although primary THA is the treatment of choice in the elderly.
